# T22-PE24-H6 Nanotoxin Selectively Kills CXCR4-High Expressing AML Patient Cells In Vitro and Potently Blocks Dissemination In Vivo

**DOI:** 10.3390/pharmaceutics15030727

**Published:** 2023-02-22

**Authors:** Yáiza Núñez, Annabel Garcia-León, Aïda Falgàs, Naroa Serna, Laura Sánchez-García, Ana Garrido, Jorge Sierra, Alberto Gallardo, Ugutz Unzueta, Esther Vázquez, Antonio Villaverde, Ramon Mangues, Isolda Casanova

**Affiliations:** 1 Institut d’Investigació Biomèdica Sant Pau (IIB SANT PAU), 08041 Barcelona, Spain; 2 Josep Carreras Leukaemia Research Institute (IJC), 08916 Badalona, Spain; 3 CIBER Bioingeniería, Biomateriales y Nanomedicina (CIBERBBN), Instituto de Salud Carlos III, 28029 Madrid, Spain; 4 Institut de Biotecnologia i de Biomedicina, Universitat Autònoma de Barcelona, 08193 Bellaterra, Spain; 5 Departament de Genètica i de Microbiologia, Universitat Autònoma de Barcelona, 08193 Bellaterra, Spain; 6 Department of Hematology, Hospital de la Santa Creu i Sant Pau, 08025 Barcelona, Spain; 7 Department of Pathology, Hospital de la Santa Creu i Sant Pau, 08025 Barcelona, Spain

**Keywords:** acute myeloid leukemia, CXCR4, targeted nanoparticle, disseminated AML mouse model

## Abstract

Despite advances in the development of targeted therapies for acute myeloid leukemia (AML), most patients relapse. For that reason, it is still necessary to develop novel therapies that improve treatment effectiveness and overcome drug resistance. We developed T22-PE24-H6, a protein nanoparticle that contains the exotoxin A from the bacterium *Pseudomonas aeruginosa* and is able to specifically deliver this cytotoxic domain to CXCR4^+^ leukemic cells. Next, we evaluated the selective delivery and antitumor activity of T22-PE24-H6 in CXCR4^+^ AML cell lines and BM samples from AML patients. Moreover, we assessed the in vivo antitumor effect of this nanotoxin in a disseminated mouse model generated from CXCR4^+^ AML cells. T22-PE24-H6 showed a potent, CXCR4-dependent antineoplastic effect in vitro in the MONO-MAC-6 AML cell line. In addition, mice treated with nanotoxins in daily doses reduced the dissemination of CXCR4^+^ AML cells compared to buffer-treated mice, as shown by the significant decrease in BLI signaling. Furthermore, we did not observe any sign of toxicity or changes in mouse body weight, biochemical parameters, or histopathology in normal tissues. Finally, T22-PE24-H6 exhibited a significant inhibition of cell viability in CXCR4^high^ AML patient samples but showed no activity in CXCR4^low^ samples. These data strongly support the use of T22-PE24-H6 therapy to benefit high-CXCR4-expressing AML patients.

## 1. Introduction

Acute myeloid leukemia (AML) is a malignant, heterogeneous group of hematological diseases caused by excessive clonal proliferation of myeloid precursor cells [1]. Over the last few years, considerable advances have been made in the disease pathogenesis as well as in the development of novel drugs specifically designed to treat AML patient subgroups with defined molecular alterations [2]. Although most AML patients initially develop chemosensitivity to multiple cycles of intensive chemotherapy and hematopoietic cell transplantation, the majority of adults have an intermediate or adverse prognosis (49% and 31%, respectively), and about two-thirds of patients relapse [3,4,5,6]. For that reason, it is still necessary to discover novel drugs that improve AML patient survival while avoiding drug resistance. 

CXCR4 is a cell-surface receptor that has been described as being overexpressed in more than 20 cancer types, including hematologic and solid neoplasias [7,8,9], and is the chemokine receptor most commonly expressed by cancer cells [10]. The interaction of the CXCR4 receptor with its ligand, CXCL12, regulates the signaling between AML cells and the bone marrow (BM) microenvironment that promotes migration and resistance to chemotherapy [7,11]. In addition, CXCR4 is highly expressed in around 50% of AML patients [12,13,14,15,16] and its overexpression correlates with a poor prognosis, which suggests that CXCR4 is an AML potential therapeutic target [12,17,18,19].

Previously, our research group developed a multivalent protein nanoparticle (T22-PE24-H6) that selectively targets CXCR4-positive leukemia cells. The T22 domain of T22-PE24-H6 interacts specifically with the chemokine receptor CXCR4 [20] and precisely delivers the most toxic virulence factor of *Pseudomonas aeruginosa*, the exotoxin A, to target cells [21]. In recent studies, we reported that T22-PE24-H6 produces a potent antitumor effect in other mouse models of different cancer types [22,23,24,25,26].

The aim of this study is to determine the antineoplastic effect of the nanoparticle T22-PE24-H6 in AML. Firstly, we report that T22-PE24-H6 has the potential to selectively eliminate CXCR4^+^ AML human cells through the induction of apoptotic death. Moreover, we demonstrate that T22-PE24-H6 treatment potently reduces leukemia burden in the BM and liver with no associated toxicity in a CXCR4^+^ disseminated AML model. Finally, we also observed a potent and selective effect of the nanoparticle in AML patients’ BM samples with elevated expression of CXCR4. These findings suggest that T22-PE24-H6 is a new CXCR4-targeted therapy that could improve outcomes for patients with AML.

## 2. Methods

### 2.1. AML Cell Lines and Patient Samples

Human leukemia cell lines OCI-AML-3, MONO-MAC-6, and HEL were purchased from DSMZ with a corresponding authentication certificate (DSMZ, Braunschweig, Germany). MONO-MAC-6 and HEL cell lines were cultured in RPMI-1640 medium, whereas OCI-AML-3 was cultured in α-MEM medium (Gibco, Thermo Fisher Scientific, Waltham, MA, USA). All cell lines were supplemented with 10% FBS, 2 mM L-glutamine, and 100 U/mL penicillin/streptomycin (Gibco, Thermo Fisher Scientific), and were incubated at 37 °C with 5% CO_2_. The MONO-MAC-6 cell line was also supplemented with 1.0 mM sodium pyruvate, 10 µg/mL insulin, and 0.1 mM non-essential amino acids (Gibco, Thermo Fisher Scientific).

Primary AML BM samples (*n* = 10) were collected at diagnosis after obtaining written informed consent in accordance with the Declaration of Helsinki and with the approval of the Clinical Research Ethics Committee of our institution (Project code: IIBSP-CXC-2014-86; Committee Code: 03/2015). The main patient characteristics are shown in Table 1. Mononuclear cells were isolated by lymphoprep gradient centrifugation (StemCell Technologies, Vancouver, Canada) and stored in liquid nitrogen after controlled freezing. For proliferation assays, flow cytometry (FC), and immunohistochemistry (IHC) analyses, cells were thawed, and red blood cells were lysed with RBC lysis buffer (Invitrogen Thermo Fisher Scientific). Cells were counted and incubated at 37 °C with 5% CO_2_ in IMDM supplemented with 3% heat-inactivated FBS, 2 mM L-glutamine (Gibco, Thermo Fisher Scientific), 20% BIT 9500 Serum Substitute (StemCell Technologies), 5 ng/mL IL-3 (PeproTech, Cranbury, NJ, USA), 5 × 10^−5^ M β-mercaptoethanol (Sigma-Aldrich, Saint Louis, MO, USA), 1.0 mM sodium pyruvate, and 0.1 mM non-essential amino acids (Gibco, Thermo Fisher Scientific).

### 2.2. Lentiviral Luciferase Transduction

MONO-MAC-6-Luci cell lines were obtained by lentiviral transduction with the plasmids pLenti-III-UbC-luc as described in previous work [24]. The cell line was selected in medium containing 2 µg/mL puromycin (InvivoGen, San Diego, CA, USA) for 3 weeks until stable clones were obtained.

### 2.3. Nanoparticle Design and Production

The T22-PE24-H6 protein monomers of ~6 nm (29.2 kDa) are formed by a T22 domain at the N terminus that targets CXCR4^+^ cancer cells, followed by a cytotoxic domain, which is the de-immunized catalytic domain of the *Pseudomonas aeruginosa* exotoxin A (PE), and the polyhistidine tag (H6) at the C-terminus, which triggers self-assembly to form a toroidal multimeric T22-PE24-H6 nanoproteins of ~60 nm. The T22-PE24-H6 nanoarchitecture, production, and purification have been described in previous works [20,22,27].

### 2.4. Detection of CXCR4 Expression in AML Cell Lines

CXCR4 surface expression in AML cell lines was determined by fluorescence-activated cell sorting (FACS) analysis as previously described [28].

Additionally, IHC staining of CXCR4 expression was performed in paraffin-embedded cell blocks of OCI-AML-3, MONO-MAC-6, and HEL cell lines, using an anti-human CXCR4 antibody (1:200, ab124824, Abcam, Cambridge, UK) in an Autostainer Link 48 (Agilent Technologies, Santa Clara, CA, USA), following the manufacturer’s instructions. Representative images were taken using an Olympus DP73 digital camera (Olympus, Tokyo, Japan) at an original magnification of ×400 and processed with Olympus CellD Imaging 3.3 software.

### 2.5. Analysis of T22-PE24-H6 Antineoplastic Effect In Vitro

A cell viability assay was used to assess the antineoplastic activity of T22-PE24-H6 in AML cell lines. Cells were cultured at 2.5 × 10^5^ cells/mL for 24 h in 96-well plates and were treated with T22-PE24-H6 at 2, 10, and 50 nM or buffer (166 mM NaCO_3_H, pH = 8). After 48 h of incubation, a Cell Proliferation Kit II (Hoffmann-La Roche, Basel, Switzerland) was used according to the manufacturer’s instructions. Five technical and three biological replicates were performed. The half-maximal inhibitory concentration (IC_50_) was calculated using non-linear regression algorithms and a four-parameter logistic model with log-transformed data for each AML cell line using GraphPad Prism 8.0.1 (GraphPad Software, San Diego, CA, USA).

To evaluate the CXCR4-specific antineoplastic activity of T22-PE24-H6, a receptor competition assay was performed with the CXCR4 antagonist, AMD3100 (Sigma Aldrich, St. Louis, MI, USA). The in vitro antineoplastic activity of 10 nM T22-PE24-H6 was determined in MONO-MAC-6 cells with or without a 1 h pre-treatment with AMD3100 at 100 nM.

To find out the cell death mechanism, nuclear staining was performed with 4′-6-diamidino-2-phenylindole (DAPI) dye in MONO-MAC-6 cells (2.5 × 10^5^ cells/mL) treated with buffer or 10 nM T22-PE24-H6 for 24 and 48 h, as previously described [29]. Apoptosis events were quantified using a cell counter tool in Image J Fiji software v.1.8.0.172 (NIH, Bethesda, MD, USA) in six random fields of each condition.

Additionally, an Annexin V/PI staining assay (Annexin V-FITC Apoptosis Detection Kit, Sigma Aldrich) was performed in MONO-MAC-6 cells at the same experimental conditions. Apoptotic cells were quantified by FC using a MACSQuant Analyzer 10 flow cytometer, and data were analyzed by MACS Quantify 2.13.1 software (Miltenyi Biotec, Bergisch Gladbach, Germany).

Furthermore, IHC staining of cleaved caspase-3 and cleaved PARP was performed in paraffin-embedded cell blocks from MONO-MAC-6 that had been treated with 10 nM of T22-PE24-H6 or buffer for 24 or 48 h. Images were taken using Pannoramic SCAN II (3DHISTECH, Budapest, Hungary) at an original magnification of 400 and processed with Image J Fiji software v.1.8.0.172 (NIH), choosing eight fields randomly for each condition.

### 2.6. Evaluation of T22-PE24-H6 Antineoplastic Activity in a Disseminated AML Mouse Model

In vivo procedures were conducted in accordance with the guidelines approved by the Animal Ethics Committee of the Hospital de Sant Pau (project number 10108 by the Government of Catalonia) and performed following the European Union Directive 2010–63-EU for the welfare of laboratory animals. Four-week-old female NSG mice (NOD.Cg-Prkdc^scid^ Il2rg^tm1WjI^/SzJ) were obtained from Charles River Laboratories (Wilmington, MA, USA) and housed in microisolator units with sterile food and water ad libitum. Mice were intravenously (i.v.) injected with luciferase-labeled MONO-MAC-6 cells (MONO-MAC-6-Luci; cells 1 × 10^6^ cells/200 μL) and, two days later, were divided randomly into two groups. The buffer group (BUFFER; *n =* 11) was i.v. injected with 166 mM NaCO_3_H pH = 8 and the T22-PE24-H6 group (T22-PE24-H6; *n* = 10) with 5 μg of T22-PE24-H6 nanoparticle. Both animal groups were treated with a total of 10 daily doses. Bioluminescence imaging was used to monitor AML dissemination every 2 or 3 days using the IVIS Spectrum Imaging System (PerkinElmer, Waltham, MA, USA). Mice were weighed and euthanized by cervical dislocation under isoflurane anesthesia on the first day on which any mouse presented relevant signs of disease. The main organs and tissues were collected, fixed in 3.7% formaldehyde solution, and paraffin-embedded for further histological and IHC analyses. Additionally, blood was obtained by intracardiac extraction to evaluate treatment toxicity. Results were expressed as bioluminescence intensity (BLI) and total flux (photons/second; radiance photons) ± SEM.

### 2.7. Immunohistochemical Analysis of CXCR4 and CD45 in AML Infiltrated Tissues

IHC analysis was performed in paraffin-embedded tissue samples using CD45 antibodies (IR75161-2, Agilent Technologies) and CXCR4 antibodies (ab124824, Abcam). IHC staining was performed in an Autostainer Link 48 (Agilent Technologies), following the manufacturer’s instructions. AML organ infiltration by CD45 and CXCR4 expression was evaluated and quantified using an Olympus BX53 microscope and Image J Fiji software v.1.8.0.172 (NIH), choosing six fields randomly for each tissue. The results were expressed as stained total area ± SEM.

### 2.8. Toxicity Analysis in Mouse Tissues and Blood

All organs were analyzed histopathologically after hematoxylin-eosin (H&E) staining by two independent observers to analyze the potential toxicity of T22-PE24-H6 using an Olympus BX53 microscope.

In addition, to evaluate toxicity in the liver and kidneys after treatment, plasma aspartate transaminase (AST) and alanine transaminase (ALT) enzyme activities, as well as uric acid and creatinine levels, were determined using commercial kits (20764949322, 20764957322, 03183807190, and 04810716190, respectively. Hoffmann-La Roche) adapted for a COBAS 6000 autoanalyzer (Hoffmann-La Roche).

### 2.9. Detection of CXCR4 Expression in AML Patients’ BM Cells

CXCR4 expression in BM samples was evaluated by FACS analysis. After thawing and red blood cell lysis, 2.5 × 10^5^ mononuclear cells were incubated for 20 min at 4 °C in the dark with PE-Vio770 anti-human CXCR4 or PE-Vio770 anti-human IgG1 as the isotype control (130-120-728 and 130-113-440, respectively, Miltenyi Biotec). Twenty thousand events were acquired from each tube using a MACSQuant Analyzer 10 flow cytometer (Miltenyi Biotec). The MACSQuantify software was used to analyze the FC data, and the results were expressed as the median fluorescence intensity ratio between CXCR4 and isotype-labeled samples (MFIR) ± SEM. The median CXCR4 MRFI in BM samples was 4.67 (1.08–53.17). Using this cut-off threshold, we categorized patients into CXCR4^low^ and CXCR4^high^ expression groups.

The CXCR4 surface expression of AML patient samples was also evaluated by IHC staining. Six representative images of every patient sample were quantified as the average of all DAB staining intensities normalized by nuclear number.

### 2.10. Evaluation of T22-PE24-H6 Antineoplastic Activity in Cultured AML Patients’ BM Cells

Mononuclear cells were plated in triplicate in a 96-well plate at 5 × 10^5^ cells/mL. Each AML primary cell sample was treated with buffer or 1, 10, and 100 nM T22-PE24-H6 and incubated for 48 h at 37 °C. Then, plates were analyzed using a Cell Proliferation Kit II as described above.

### 2.11. Statistical Analysis

Differences between the two groups were analyzed using an unpaired, two-tailed Student’s t-test. When the data were not normally distributed, the comparison of variables was performed with the Mann–Whitney U-test. GraphPad Prism 8.0.1 (GraphPad Software) was used for statistical analysis. Graphs and error bars represent the mean ± SEM of independent biological experiments unless stated otherwise. Differences were considered statistically significant at *p* ≤ 0.05. All in vitro studies were performed in triplicate.

## 3. Results

### 3.1. T22-PE24-H6 Antineoplastic Effect in CXCR4^+^ AML Cell Lines

**Figure 1:**
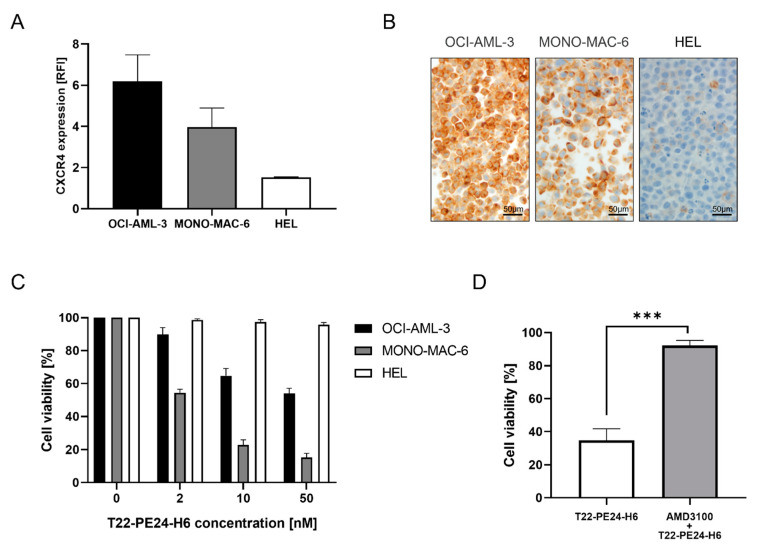
Determination of CXCR4 expression intensity and T22-PE24-H6 cytotoxic effect in AML cell lines. (**A**) CXCR4 membrane expression of AML cell lines by flow cytometry. Data acquisition was analyzed by Cell Quest Pro software, and results are expressed as RFI normalized by the isotype. (**B**) IHC analysis of CXCR4 expression in OCI-AML-3, MONO-MAC-6, and HEL cell blocks. (**C**) Evaluation of the antineoplastic activity of T22-PE24-H6 (0, 2, 10, and 50 nM) after 48 h of treatment in AML cell lines performed by the XTT assay. (**D**) Competition assays with the preincubation of AMD3100 followed by the T22-PE24-H6 addition (100 nM AMD3100: 10 nM T22-PE24-H6) in the MONO-MAC-6 cell line. An unpaired t test was used to test the differences between the groups indicated by *** when the *p*-value was ≤ 0.005. The results are expressed as mean ± SEM. Original magnification ×400 for panel B. AML: acute myeloid leukemia; RFI: relative fluorescence intensity.

We evaluated CXCR4 membrane levels in OCI-AML-3, MONO-MAC-6, and HEL cell lines by FC (Figure 1A) and IHC (Figure 1B). OCI-AML-3 and MONO-MAC-6 cell lines showed elevated levels of CXCR4 expression, while the HEL cell line had negligible levels of CXCR4 expression (OCI-AML-3: 6.2 ± 1.3 of RFI; MONO-MAC-6: 4.0 ± 0.9 of RFI; HEL: 1.5 ± 0.0 of RFI).

Moreover, we assessed the cytotoxic effect of T22-PE24-H6 in the three AML cell lines, correlating these results with their level of CXCR4 membrane expression. Results showed that MONO-MAC-6 cells were the most sensitive (IC_50_ 2.35 ± 1.12 nM), whereas OCI-AML-3 cells had lower sensitivity to the nanoparticle (IC_50_ > 50 nM). In fact, treatment with 10 nM T22-PE24-H6 for 48 h in MONO-MAC-6 and OCI-AML-3 cell lines decreased proliferation compared to buffer-treated cells. On the other hand, T22-PE24-H6 exposure showed no effect on cell viability in the HEL cell line (IC_50_ > 50 nM) due to the lack of CXCR4 membrane expression (Figure 1C).

Additionally, we studied if the T22-PE24-H6 cytotoxic effect was CXCR4-dependent. In this context, cell pre-treatment with AMD3100 significantly decreased the cell death of the MONO-MAC-6 cell line as compared to cells only exposed to T22-PE24-H6 (Figure 1D).

### 3.2. T22-PE24-H6 Induced Apoptosis in MONO-MAC-6 Cells

**Figure 2:**
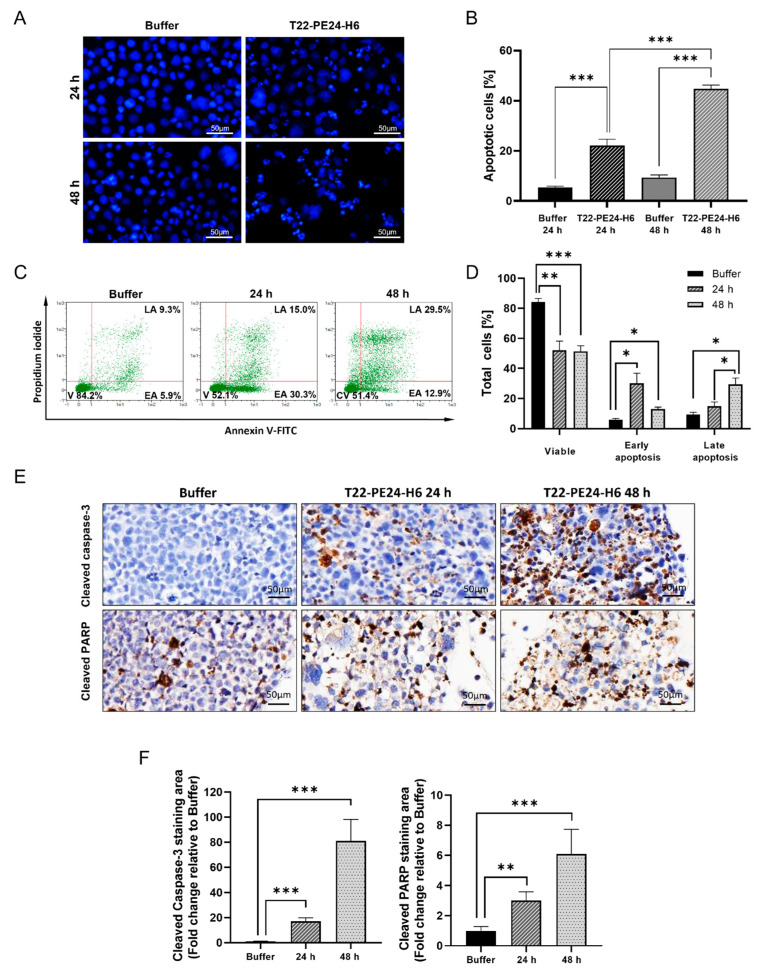
T22-PE24-H6 apoptosis induction in the CXCR4^+^ MONO-MAC-6 AML cell line. (**A**) Representative images showing DAPI-stained cell nuclei of MONO-MAC-6 AML cells and (**B**) quantification of apoptotic cells after 24 h or 48 h exposure to buffer or 10 nM T22-PE24-H6 nanoparticles. (**C**) A representative dot-plot showing the annexin V/PI assay and (**D**) quantification of viable cells (V), early apoptosis cells (EA), and late apoptosis cells (LA) after 24 h or 48 h exposure to buffer or 10 nM T22-PE24-H6 in MONO-MAC-6 cells. (**E**) IHC staining using cleaved caspase-3 and cleaved PARP antibodies after buffer or 10 nM T22-PE24-H6 treatment (24 and 48 h) in MONO-MAC-6 cells. (**F**) Quantification of the IHC positive area occupied by cleaved caspase-3 staining or cleaved PARP staining in T22-PE24-H6-treated samples as compared to buffer-treated samples (24 and 48 h) in 8 microscopic fields. The results are expressed as mean ± SEM. * *p* ≤ 0.05, ** *p* ≤ 0.01, *** *p* ≤ 0.005. The percentages in panel C are related to the mean of three independent experiments. Original magnification ×400 for panels A and E. PI: propidium iodide; PARP: poly (ADP-ribose) polymerase.

To determine if the cell death produced by treatment with T22-PE24-H6 was caused by apoptotic induction, nuclear morphological changes were studied by confocal microscopy using nuclear staining with DAPI dye (Figure 2A). MONO-MAC-6 cells treated with the nanoparticle showed clear apoptotic features that became more apparent with increasing incubation time. Apoptosis events significantly increased after 24 h of incubation with T22-PE24-H6 compared to the number of apoptotic cells in buffer-treated cells (22.1 ± 2.6 vs. 5.4 ± 0.5 apoptotic cells). After 48 h, the number of apoptotic cells treated with the nanoparticle increased significantly compared to buffer-treated cells at the same time (44.7 ± 1.5 vs. 9.4 ± 1.0 apoptotic cells) and also compared to 24 h T22-PE24-H6 treated cells (22.1 ± 2.6 apoptotic cells) (Figure 2B).

To further quantify the percentage of apoptosis in MONO-MAC-6 cells treated with T22-PE24-H6, an Annexin V/PI assay was performed. A representative result of FC is presented in Figure 2C. Twenty-four hours after treatment, the percentage of viable cells was reduced from 84.2 ± 2.4% in cells treated with buffer to 52.1 ± 6.1% in cells treated with T22-PE24-H6. The percentage of cells in early apoptosis increased from 5.9 ± 0.9% in buffer-treated cells to 30.3 ± 6.6% at 24 h and then decreased to 12.9 ± 1.3% at 48 h. Conversely, we identified an increase in late apoptosis from 9.3 ± 1.5% in cells treated with buffer to 15.0 ± 2.8% in nanoparticle-treated cells at 24 h and 29.5 ± 4.2% at 48 h (Figure 2D).

For additional validation of the apoptosis occurring in MONO-MAC-6 cells after T22-PE24-H6 treatment, a caspase-3 and a PARP IHC assay were performed. Cells treated with the nanoparticle achieved a significant 17.09-fold and 81.05-fold increase in cleaved caspase-3 positive area at 24 h and 48 h, respectively, compared to cells treated with buffer. Additionally, the positive area of cleaved PARP was also significantly higher in cells treated with T22-PE24-H6—3.01-fold at 24 h and 6.01-fold at 48 h—compared to the buffer group (Figure 2E,F). These observations indicate that T22-PE24-H6 induced apoptosis in MONO-MAC-6 cells.

### 3.3. Antineoplastic Effect of T22-PE24-H6 in a CXCR4^+^ AML Mouse Model

Considering T22-PE24-H6 nanoparticles’ potent antineoplastic effect in vitro, we assessed the anticancer activity in a disseminated AML mouse model generated by intravenous injection of MONO-MAC-6-Luci cells. The experimental design is detailed in the Section 2 and schematized in Supplemental Figure S1A.

**Figure 3:**
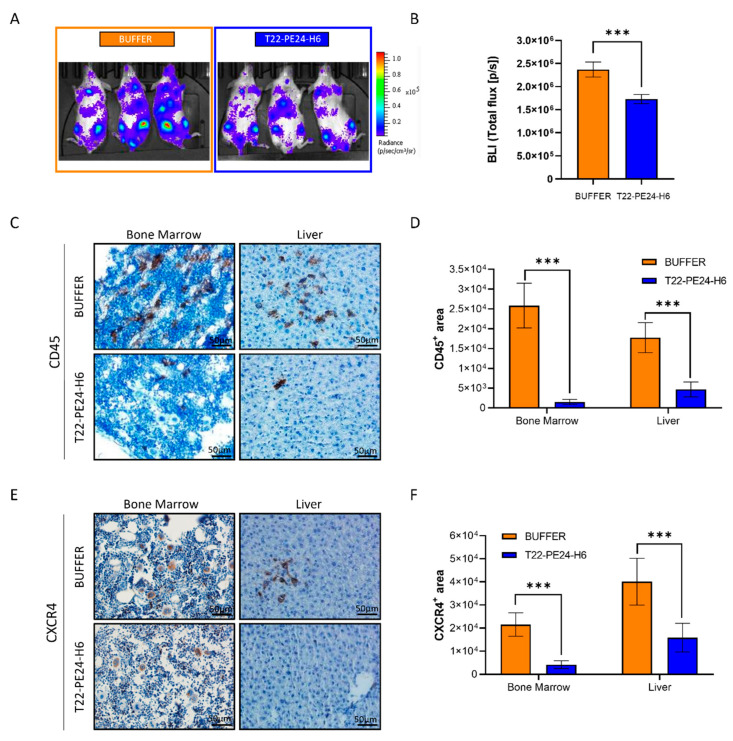
T22-PE24-H6 antineoplastic effect in a CXCR4^+^ AML disseminated mouse model. (**A**) Representative bioluminescent images of mice treated with buffer (NaCO3H + NaCl pH = 8) or 5 µg T22-PE24-H6 after 10 doses. (**B**) BLI at day 12 after MONO-MAC-6-Luci cell injection, comparing the BUFFER and T22-PE24-H6 mouse groups. (**C**) Anti-human CD45 staining representative images, and (**D**) quantification of leukemic cells in the BM and liver of mice treated with buffer or T22-PE24-H6. (**E,F**) Detection and quantification of CXCR4 levels, by IHC, in the BM and liver of the leukemic mice 14 days after i.v. injection of MONO-MAC-6-Luci cells. The results are presented as mean of staining area ± SEM. Statistical analysis was performed using the T-test or Mann-Whitney *U* test, and significant differences between the groups are indicated as *** *p* ≤ 0.005. Original magnification ×400 for panels A and C. BLI: bioluminescence intensity.

The results showed that treatment with T22-PE24-H6 nanoparticles significantly reduced mouse BLI compared to buffer-treated mice after ten doses (1.37-fold decrease), which indicates a reduction in leukemia dissemination (Figure 3A and Supplemental Figure S1B). No body weight differences between the groups were found (Supplemental Figure S1C).

Tissue samples of all organs were collected and paraffin-embedded after mouse euthanasia and used to analyze AML dissemination and toxicity. Leukemic burden was quantified by IHC with the human CD45 marker. The T22-PE24-H6 treatment significantly decreased the dissemination of leukemic cells in the BM and liver compared to buffer-treated mice (17.2 and 3.8-fold decrease, respectively) (Figure 3C,D). In addition, to further analyze the T22-PE24-H6 nanoparticle effect, CXCR4 IHC staining was also performed in the BM and liver of both animal groups. Expression of CXCR4 showed a statistically significant reduction in the T22-PE24-H6 group compared to the BUFFER group (a 5.2- and 2.5-fold decrease in the BM and liver, respectively) (Figure 3F,G). These results suggest that T22-PE24-H6 diminishes CXCR4^+^ AML cells in infiltrated organs, leading to reduced leukemic dissemination.

### 3.4. Toxicity Evaluation in Mouse Tissues

**Figure 4:**
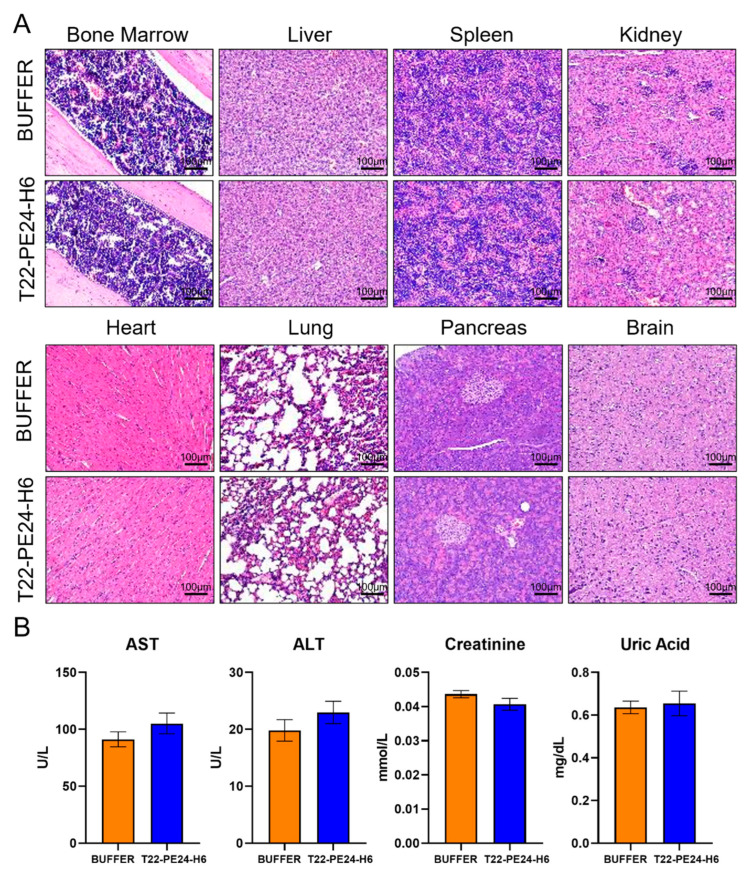
Evaluation of T22-PE24-H6-induced toxicity. (**A**) H&E staining of the BM, liver, spleen, kidney, heart, lung, pancreas, and brain after T22-PE24-H6 or buffer treatment. (**B**) AST, ALT, uric acid, and creatinine levels in plasma comparing the BUFFER and T22-PE24-H6 mouse groups. Original magnification ×200 for panel A. Statistical analysis performed by the Mann-Whitney U test. The results are expressed as mean ± SEM. AST: aspartate aminotransferase; ALT: alanine aminotransferase.

The toxicity associated with the T22-PE24-H6 treatment was assessed at biochemical and histopathological levels. Inflammatory processes and morphological changes were evaluated by H&E staining in mouse organs, including the BM, liver, spleen, kidney, heart, lung, pancreas, and brain (Figure 4A). In addition, serum concentrations of biochemical markers AST, ALT, creatinine, and uric acid were measured at the end of the experiment to evaluate liver and renal functions. No significant differences were found comparing biochemical parameters between the BUFFER and T22-PE24-H6 mouse groups (Figure 4B). Our results showed no toxicity-related alterations in all tissues studied by H&E nor in plasma samples. Therefore, these results suggest that T22-PE24-H6 treatment had a potent antineoplastic effect without inducing any alterations in the mice’s organs.

### 3.5. T22-PE24-H6 In Vitro Effect in AML Patients’ BM Samples

**Figure 5:**
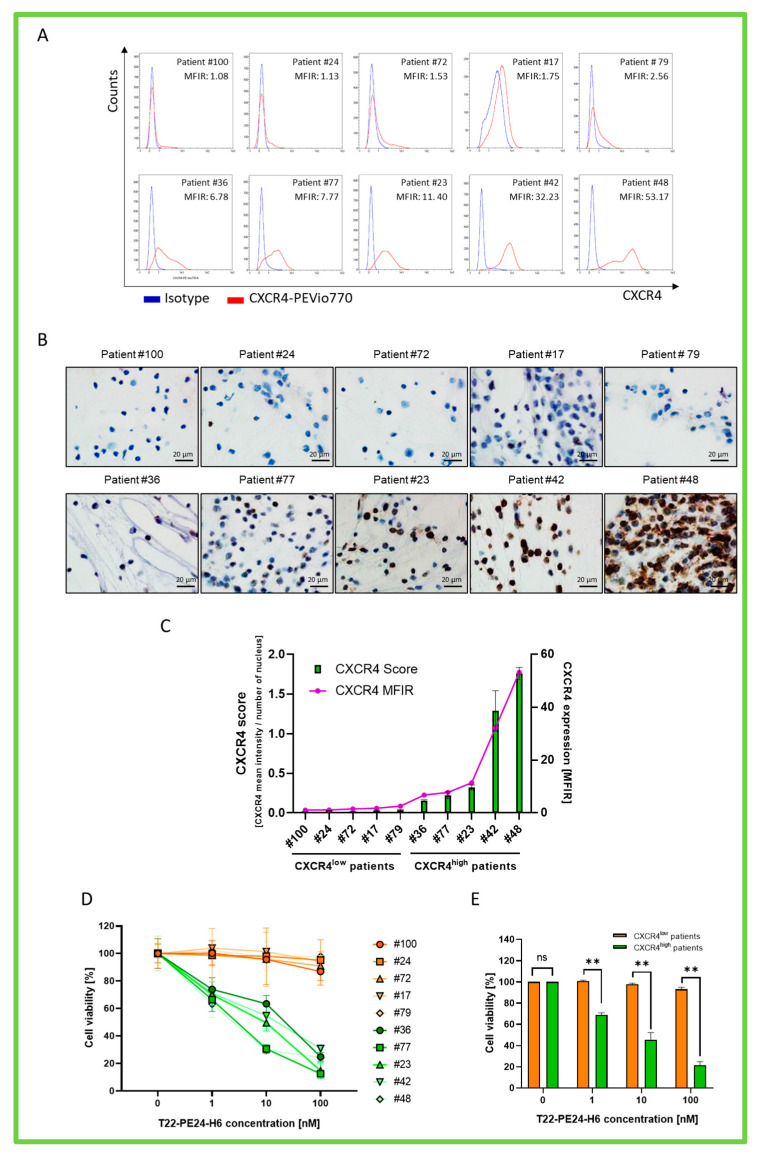
CXCR4 expression levels and T22-PE24-H6 in vitro anticancer effect in the BM AML patient samples. (**A**) Flow cytometric evaluation of CXCR4 surface expression in the BM samples using an anti-CXCR4 antibody. An isotype control antibody was used as a control. (**B**) Representative IHC staining images of CXCR4 from ten AML patient samples. (**C**) Median fluorescence intensity of surface CXCR4 expression relative to isotype control (purple line) and CXCR4 score intensity (green bars). (**D**) Individual evaluation of cell viability of ten AML patients 48 h after treatment with 0, 1, 10, or 100 nM T22-PE24-H6 (patient samples with low CXCR4 expression are represented in orange, and those with high CXCR4 expression are in green). (**E**) Evaluation of the antineoplastic activity of T22-PE24-H6 in CXCR4 low-expression AML patient samples (orange, *n* = 5) and CXCR4 high-expression AML patient samples (green, *n* = 5). Original magnification of x1000 for panel B. A Mann–Whitney U-test was used to compare mean CXCR4 expression between the CXCR4^high^ and CXCR4^low^ patient groups, as indicated by ** when the *p*-value was ≤ 0.01. MFIR: median fluorescence intensity ratio; ns: not significant.

Finally, we evaluated the antiproliferative effect of T22-PE24-H6 in primary BM samples obtained from AML patients at diagnosis. To that aim, we selected a total of 10 BM samples: 5 of them had high levels of CXCR4 expression (Patient #36: 6.78 MFIR; Patient #77: 7.77 MFIR; Patient #23: 11.40 MFIR; Patient #42: 32.23 MFIR; Patient #48: 53.17 MFIR) and the other 5 samples showed low levels of the receptor (Patient #100: 1.08 MFIR; Patient #24: 1.13 MFIR; Patient #72: 1.53 MFIR; Patient #17: 1.75 MFIR; Patient #79: 2.56 MFIR) (Figure 5A). Additionally, we confirmed the results obtained by FC by performing a CXCR4 intensity score by IHC (Figure 5B). CXCR4 expression levels showed similar results using both techniques for each tested sample (Figure 5C).

Then, we measured the cell viability of each AML patient’s primary sample by incubating them with different concentrations of the nanoparticle (1, 10, and 100 nM T22-PE24-H6) or buffer for 48 h, and then cell viability was determined as previously described in cell lines.

Results showed that T22-PE24-H6 displays potent in vitro antitumor activity only in AML patient samples showing high CXCR4 expression but no effect in BM samples with low receptor expression (Figure 5D). After dividing patient samples into two groups based on CXCR4 expression, cell viability in the CXCR4^high^ group was significantly reduced compared to the CXCR4^low^ group (45.6 ± 6.7% cell viability vs. 97.8 ± 1.0% cell viability at 10 nM of T22PE-24-H6, respectively) (Figure 5E).

## 4. Discussion

We found that exposure of different CXCR4^+^ AML cell lines to T22-PE24-H6 nanoparticles in the low nanomolar range induces a potent cytotoxic effect mediated by apoptosis, as confirmed by its characteristic features, such as DNA condensation, apoptotic body formation, externalization of phosphatidylserine residues on the outer plasma membrane, as detected by Annexin V, caspase-3 activation, and PARP proteolysis in target cancer cells. Our findings are therefore in agreement with the definition of the molecular activation of apoptotic cell death [30] and some previous studies, reported by other researchers, concerning the induction of apoptosis by PE [31,32].

Indeed, T22-PE24-H6 showed in vitro CXCR4-dependent cytotoxicity in human AML cell lines, as shown by the competition assay with AMD3100 and the lack of sensitivity to the nanotoxin in the CXCR4^-^ cell line. However, despite the OCI-AML-3 cell line expressing higher levels of membrane CXCR4 compared to the MONO-MAC-6 cell line, it showed lower sensitivity to T22-PE24-H6. In fact, the cells’ sensitivity to the nanotoxin is not only dependent on the level of CXCR4 expression but also on the sensitivity to the exotoxin itself. It is described in the literature that the proapoptotic Bak protein is required to induce apoptosis by *Pseudomonas aeruginosa* exotoxin A and that the overexpression of the antiapoptotic factor Mcl-1 inhibits PE-induced death [33]. Interestingly, the MONO-MAC-6 cell line expresses higher levels of BAK and lower levels of Mcl-1 compared to the OCI-AML-3 cell line [34,35]. Thus, we suggest that the different levels of expression of the Bcl-2 family proteins could explain the higher sensitivity of the MONO-MAC-6 cells to the nanotoxin compared to the OCI-AML-3 cells.

Moreover, our results showed that repeated intravenous injections of this nanotoxin blocked CXCR4^+^ leukemic cell dissemination in the BM and liver in a CXCR4^+^ AML mouse model without causing on-target or off-target toxicity. Importantly, cultured cells derived from the BM samples of AML patients that highly express CXCR4 are sensitive to this nanoparticle. Taken together, these results suggest that T22-PE24-H6 could be developed as a novel treatment applicable to around 50% of AML patients who overexpress CXCR4, especially when considering that high CXCR4 expression is associated with a poor prognosis in AML patients [12,13,14,15,16].

Our protein-only nanoparticle exploits a targeted drug delivery strategy that uses a CXCR4 ligand, named T22, to interact specifically with this receptor to trigger its internalization by endocytosis in target AML cells, an event confirmed by endosomal formation followed by subsequent endosomal escape [20,22]. Then, the exotoxin A domain of *Pseudomonas aeruginosa* is released from the T22 ligand through furin-cleavage sites and delivered in the cytosol, inhibiting protein synthesis and inducing cell death by inactivating Eukaryotic elongation factor 2 (eEF-2) [30]. Thus, cell death induction in target AML cells is due to the effect of exotoxin A domain delivery in the CXCR4^+^ cell cytosol. In contrast, the T22 ligand and the additional nanoparticle sequences are not involved in cell death induction since their role is to transport the exotoxin A.

This is radically different from previously reported nanoparticles or small molecules that were designed as CXCR4 antagonists [36,37]. Thus, their anticancer activity is due to a direct interaction with the receptor, leading to the inhibition of survival and proliferative pathways downstream of CXCR4 that maintain the cancer state. Hence, CXCR4 antagonists are considered molecularly targeted drugs, clearly different from targeted drug delivery approaches.

The drug delivery strategies closest to our CXCR4-targeted nanotoxin include polymeric nanomedicines and antibody-drug conjugates (ADCs). The first is a gene therapy approach that uses nanoparticles based on cholesterol-modified plerixafor that target CXCR4^+^ BM cells and deliver in their cytosol siRNA against Runx1 to induce their selective killing [38]. However, this work describes only in vitro cytotoxicity in BM cells obtained from an AML mouse model that displays high CXCR4 and Runx1 expression. It would be necessary to analyze their biodistribution and antitumor activity in vivo to evaluate their translational capacity.

Additional targeted drug delivery systems based on ADCs have been marketed for the therapy of hematological neoplasms [39], none of which target CXCR4 cancer cells. Nevertheless, two CXCR4-targeted ADCs that transport site-directed conjugated Auristatin E are in preclinical assays. One blocks AML dissemination in different mouse models; however, it is associated with on-target and off-tumor toxicity in BM, showing a reduction in CXCR4^+^ granulocyte-monocyte progenitors and erythroblasts and a decrease in neutrophils and monocytes in the blood [40]. Another study demonstrates a reduction in lung metastases in an osteosarcoma model, again associated with a decrease in hematopoietic stem cell populations in ADC-treated mice [41]. None of these ADCs have entered clinical trials, most likely because of concerns regarding on-target and off-tumor toxicity.

In order to overcome these limitations, our group employed several strategies to develop therapeutic nanoparticles based on the structure of T22-GFP-H6, either through the conjugation of the nanocarrier with cytotoxic drugs or through the incorporation of toxin domains in its structure, as well as pro-apoptotic proteins (PUMA, BAX, BAK) [42], or toxins from bacteria, such as PE24 or DITOX from *C. diphtheridae* [22], from animals, such as snake venom or gomesin from spiders [43], or vegetables, such as ricin [44].

Particularly, T22-PE2-H6 and T22-DITOX-H6 toxins have special interest because of their potent cytotoxic effect at low nanomolar concentrations in both dividing and non-dividing cells. Killing quiescent AML cells is important for therapy since quiescent leukemic stem cells (LSCs) are responsible for resistance to current therapy, and CXCR4+ AML cells are considered LSCs.

Additionally, in contrast to previous publications, intravenously repeated T22-PE24-H6-treated mice showed no functional or histological alterations, being indistinguishable from buffer-treated animals. Similarly, we did not find any toxicity in the spleen, an organ that also accumulates cells with low CXCR4 expression. Finally, we also observed a lack of off-target toxicity in normal organs that do not express CXCR4, such as the liver, kidney, heart, lung, pancreas, or brain.

Similarly to ADCs, therapy with immunotoxins (ITs), consisting of a targeting antibody bound to a bacterial toxin, is associated with off-target dose-limiting toxicities [45] because of unstable linkages that prematurely release the toxin and the induction of immunogenicity by the incorporated nonhuman proteins [46]. Thus, the marketed Moxetumomab pasudotox-tdfk (carrying a PE38 domain) induces hemolytic uremic syndrome and capillary leak syndrome [47]. In this context, we instead accommodated the de-immunized PE24 domain in our nanotoxin to avoid the severe side effects observed in PE38-based IT therapy [48,49].

Moreover, the already marketed targeted drug delivery products use bivalent monoclonal antibodies for targeting and show dose-limiting toxicities that bring on a narrow therapeutic window. In contrast, our novel targeting approach instead uses a multivalent protein nanoparticle that greatly increases its therapeutic window as compared to ADCs and ITs. We propose that this improvement is likely due to the multivalence of the T22-PE24-H6 nanoparticle, owed to its design that incorporates cationic T22 and H6 domains that trigger its oligomerization to display multiple T22 ligands that interact with CXCR4 receptors [22]. This is a similar structure to that of T22-GFP-H6 nanoparticles that bear the same domains in the C- and N-termini and exhibit eleven T22 ligands per nanoparticle [50], as compared to two ligands for each ADC or IT.

Remarkably, multivalency confers the capacity to selectively internalize in CXCR4-overexpressing (CXCR4^+^) cells, showing a dramatically high uptake in CXCR4^+^ cancer tissues that reaches around 80% of the injected dose (%ID), with negligible distribution in normal tissues [51,52]. In contrast, ADCs reach only 0.01%ID in tumor tissues, whereas most of the dose accumulates in the liver [53].

T22-PE24-H6 therapy would be appropriate for the 50% of AML patient candidates whose leukemic cells overexpress CXCR4, a feature that confers a poor prognosis and higher relapse rate [12,14,16,18]. This is because CXCR4 signaling is essential to maintain the leukemic stem cell phenotype, playing a crucial role in the retention and protection of leukemic cells in the BM niche, which is associated with resistance and relapse that two-thirds of chemotherapy-treated AML patients experience [7,11,54,55,56].

Thus, we believe that this nanoparticle could be used to treat CXCR4^+^ refractory or relapsed AML that becomes resistant to chemotherapy. Our nanotoxin therapy could be best applied to relapsed patients that have residual LSCs in the BM that are resistant and non-cycling, since T22-PE24-H6 is capable of selectively killing CXCR4^+^ quiescent leukemic blasts. This is consistent with the highly significant reduction induced in BM leukemic burden after nanotoxin treatment in vivo, using the same mechanism of action as ITs that kill non-dividing cells by inhibiting eEF-2 and protein translation synthesis [57]. Therefore, in contrast to the use of CXCR4 antagonists that mobilize AML cells from the BM to start dividing to be subsequently treated with chemotherapy, our approach is capable of directly killing quiescent AML in the BM niche.

In conclusion, repeated intravenous administration of T22-PE24-H6 achieves a potent antineoplastic effect in a CXCR4^+^ AML mouse model by inducing a large reduction in leukemic cells in the BM and liver. Importantly, this anticancer activity is achieved without any toxicity. Our results validate CXCR4 overexpressing cells as a relevant clinical target in AML patients; the assessment of this marker would be required to include candidate patients to be treated with our novel targeted delivery approach.

## Figures and Tables

**Table 1:** Clinical and laboratory characteristics of 10 de novo AML patients.

Patient No.	Sex	Age (yr)	FAB	2016 WHO Classification	Cytogenetics	FLT3 Mutation	NPM1 Mutation	CEBPA Mutation	CXCR4 MFIR
#100	M	18	M1	AML with biallelic mutation of CEBPA	Normal	Negative	Negative	Positive	1.08
#24	M	38	M5	AML with t(8;21)(q22;q22.1); *RUNX1*-*RUNX1T1*	45,X,Y,t(8;21)(q22;q22) [17]/46,XY [3]	Negative	Negative	Negative	1.13
#72	M	26	M4	AML with biallelic mutation of CEBPA	Normal	Negative	Negative	Positive	1.53
#17	F	71	M2	AML with mutated *NPM1*	Normal	ITD	Positive	Negative	1.75
#79	F	61	M4	AML with mutated *NPM1*	Normal	ITD	Positive	Negative	2.56
#36	F	45	M5	AML with t(9;11)(p21.3;q23.3; MLLT3-KMT2A	46,XX,t(9;11) (p22;q23) [20]	Negative	Negative	Negative	6.78
#77	M	71	M5	AML with myelodysplasia-related changes	Normal	Negative	Negative	Negative	7.77
#23	F	80	M4	AML with mutated *NPM1*	Normal	Negative	Positive	Negative	11.40
#42	F	66	M4	AML with mutated *RUNX1*	Normal	TKD	Negative	Negative	32.23
#48	M	79	M5	AML with myelodysplasia-related changes	Normal	Negative	Positive	negative	53.17

Patient No.: patient number; yr: years; FAB: French-American-British classification; FLT3: FMS-related tyrosine kinase 3; NPM1: nucleophosmin 1; CEBPA: CCAAT/enhancer-binding protein alpha; MFIR: median fluorescence intensity ratio.

## Data Availability

For original data, please contact rmangues@santpau.cat.

## Supplementary Materials

The following supporting information can be downloaded at: https://www.mdpi.com/article/10.3390/pharmaceutics15030727/s1. Figure S1. Experimental design and evolution of body weight of mice during the study of antineoplastic effect of T22-PE24-H6. (A) Schematic diagram of in vivo experimental design. (B) Follow-up of AML dissemination of mice treated with buffer or T22-PE24-H6 over the time. (C) Mean body weights of mice exposed to repeated doses T22-PE24-H6 or buffer over 10 doses. Statistical analysis was performed using T-test or Mann-Whitney U test and significant differences between groups are indicated as *** *p* ≤ 0.005
