# Supplementary material for: T22-PE24-H6 Nanotoxin Selectively Kills CXCR4-High Expressing AML Patient Cells In Vitro and Potently Blocks Dissemination In Vivo
Source: Pharmaceutics. 2023 Feb 22;15(3):727. doi: 10.3390/pharmaceutics15030727 (PMC10054149; doi:10.3390/pharmaceutics15030727)
Supplement: Supplementary file 1 [file pharmaceutics-15-00727-s001.zip › pharmaceutics-2200926-supplementary.pdf]

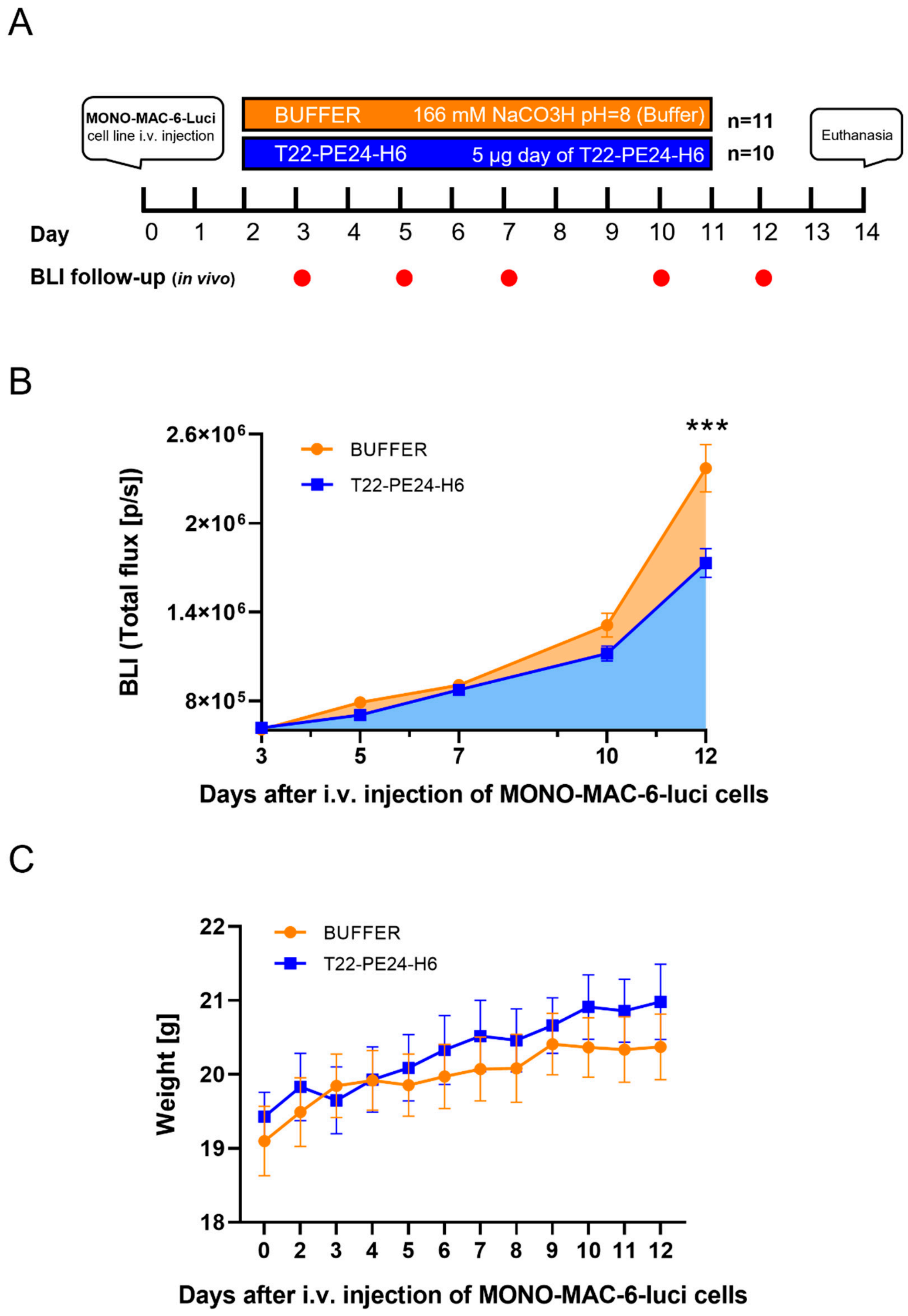

Supplemental Figure S1. Experimental design and evolution of body weight of mice during the study of antineoplastic effect of T22-PE24-H6. (**A**) Schematic diagram of *in vivo* experimental design. (**B**) Follow-up of AML dissemination of mice treated with buffer or T22-PE24-H6 over the time. (**C**) Mean body weights of mice exposed to repeated doses T22-PE24-H6 or buffer over 10 doses. Statistical analysis was performed using T-test or Mann-Whitney *U* test and significant differences between groups are indicated as ***$p \leq 0.005$.
